# Exotic urban trees conserve similar natural enemy communities to native congeners but have fewer pests

**DOI:** 10.7717/peerj.6531

**Published:** 2019-03-07

**Authors:** Steven D. Frank, Kristi M. Backe, Casey McDaniel, Matthew Green, Sarah Widney, Robert R. Dunn

**Affiliations:** 1 Department of Entomology & Plant Pathology, North Carolina State University, Raleigh, NC, USA; 2 Clemson University Arthropod Collection, Clemson, SC, USA; 3 Department of Applied Ecology, North Carolina State University, Raleigh, NC, USA

**Keywords:** Urban trees, Parasitoids, Conservation, Pest management, Exotic trees, *Acer* spp. (maple), *Quercus* spp. (oaks), Natural enemies, Native trees, Scale insects (Coccoidea)

## Abstract

Urban trees serve a critical conservation function by supporting arthropod and vertebrate communities but are often subject to arthropod pest infestations. Native trees are thought to support richer arthropod communities than exotic trees but may also be more susceptible to herbivorous pests. Exotic trees may be less susceptible to herbivores but provide less conservation value as a consequence. We tested the hypotheses that native species in *Acer* and *Quercus* would have more herbivorous pests than exotic congeners and different communities of arthropod natural enemies. The density of scale insects, common urban tree pests, was greatest on a native *Acer* and a native *Quercus* than exotic congeners in both years of our research (2012 and 2016) and sometimes reached damaging levels. However, differences in predator and parasitoid abundance, diversity, and communities were not consistent between native and exotic species in either genus and were generally similar. For example, in 2012 neither predator nor parasitoid abundance differed among native and exotic *Acer* congeners but in 2016 a native species, *A. saccharum*, had the least of both groups. A native, *Q. phellos*, had significantly more predators and parasitoids in 2012 than its native and exotic congeners but no differences in 2016. Parasitoid communities were significantly different among *Acer* species and *Quercus* species due in each case to greater abundance of a single family on one native tree species. These native and exotic tree species could help conserve arthropod natural enemies and achieve pest management goals.

## Introduction

Urban trees serve a critical conservation function by supporting herbivorous arthropod communities that, in turn, support vertebrate and invertebrate communities at higher trophic levels (Burghardt, Tallamy & Gregory Shriver, 2009; Morse, 1971; Tallamy, 2004). However, herbivorous arthropods can also be serious pests of urban trees that reduce tree health and the ecosystem services trees provide (Raupp, Shrewsbury & Herms, 2010). Thus, a potential conflict exists between the conservation value and aesthetic value of urban trees. To balance any such tradeoff and manage urban trees for the benefit of wildlife and people, it is important to understand factors that influence the arthropod communities they support.

Cities contain many native tree species that share an evolutionary history with local herbivores and many exotic tree species that do not (Aronson et al., 2015; Raupp, Cumming & Raupp, 2006; Riley, Herms & Gardiner, 2018). The evolutionary history between plants and arthropods in a habitat is an important factor influencing herbivore diversity and herbivory (Ehrlich & Raven, 1964; Farrell, Mitter & Futuyma, 1992). The “enemy release hypothesis” predicts that, because native herbivores are not adapted to exotic plant defenses, exotic plants should have fewer herbivores and be subject to less herbivory than are native plants (Keane & Crawley, 2002). This outcome would fulfill pest management objectives, as pest damage to trees would be minimal, but would make exotic plants less useful for supporting biodiversity. Alternatively, the “biotic resistance hypothesis” predicts that exotic plants can be consumed by native herbivores against which they are not defended (Hokkanen & Pimentel, 1989; Parker & Hay, 2005). This outcome may fulfill conservation goals, as trees would support arthropod communities, but only if trees do not require insecticide applications and are not severely damaged or killed. Both hypotheses have received mixed support, with research finding that herbivore abundance or herbivory on exotic plants can be more than, less than, or equal to that of native plants (Keane & Crawley, 2002; Zuefle, Brown & Tallamy, 2008).

The composition and effects of herbivore communities supported by exotic tree species in cities are difficult to predict. Cities are relatively recent human constructions and have many unnatural characteristics to which few animal species have evolved (Grimm et al., 2008; Shochat et al., 2006). For example, the urban heat island effect can alter natural enemy communities, insect and plant phenology, and affect host tree susceptibility to herbivory (Dale & Frank, 2017, 2018; Meineke, Dunn & Frank, 2014; Raupp, Shrewsbury & Herms, 2010). In addition, many native and exotic urban tree species have chronic or outbreak pest populations that are greater than for the same species in natural areas (Conway & Vander Vecht, 2015; Long, D’Amico & Frank, 2018; Raupp, Shrewsbury & Herms, 2010). For these reasons, ecological theories developed in natural ecosystems may not predict herbivore responses in cities (Dale & Frank, 2018).

**Figure 1:**
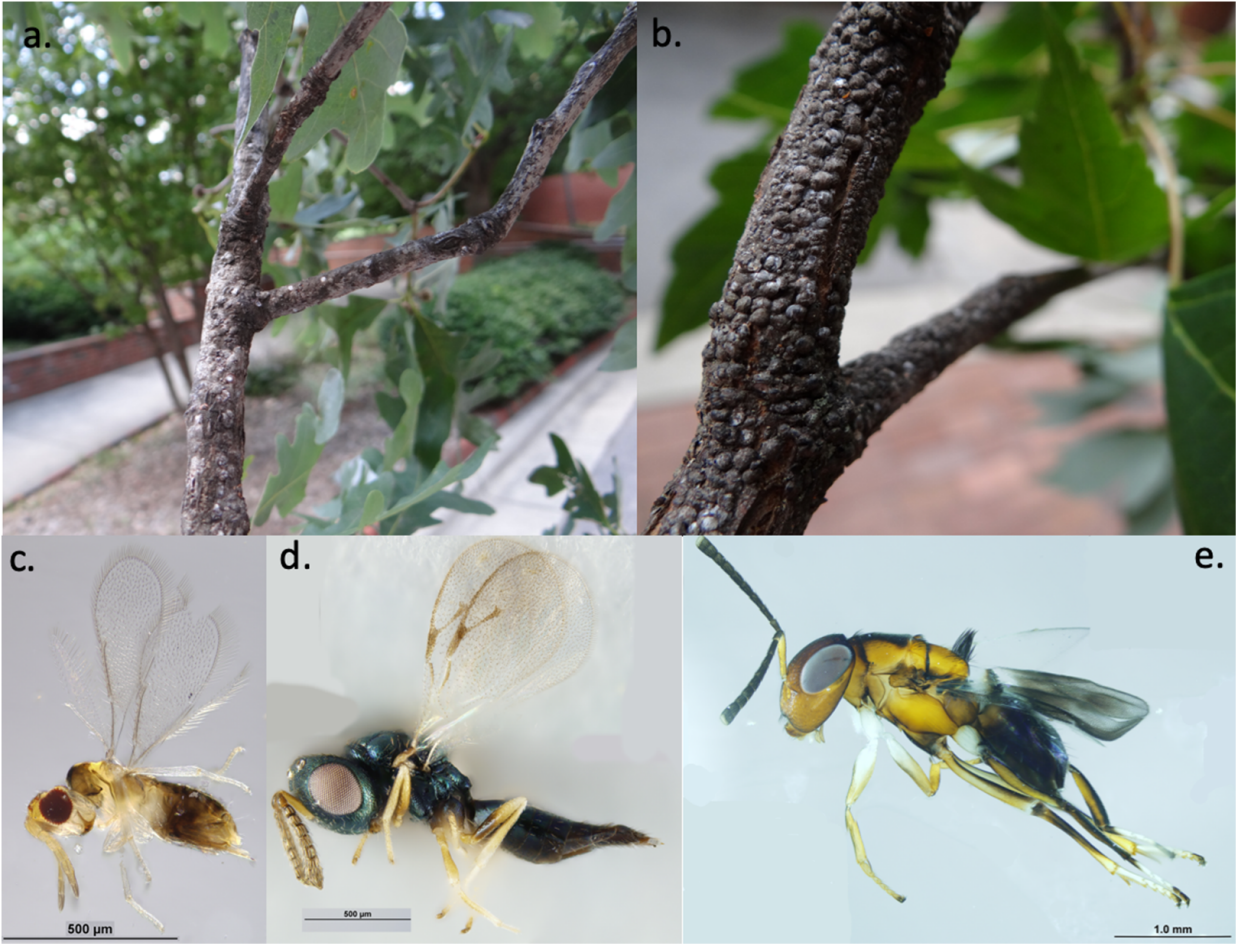
Examples of study organisms. Examples of armored scale insects (A) obscure scales (*Melanaspis obscura*) on white oak (*Q. alba*) and (B) gloomy scales (*M. tenebricosa*) on red maple (*A. rubrum*) and parasitoid wasps (C) *Encarsia* spp. (Hymenoptera: Aphelinidae), (D) *Pachyneuron* spp. (Hymenoptera: Pteromalidae), and (E) *Encyrtus* spp. (Hymenoptera: Encyrtidae) that parasitize scale insects. Photos and identifications: Andrew Ernst.

Scale insects (Hemiptera: Coccoidea) are among the most common and abundant herbivores on urban trees (Dale & Frank, 2014b; Meineke et al., 2013; Raupp, Shrewsbury & Herms, 2010; Tooker & Hanks, 2000; Wu, Jamieson & Kielbaso, 1991). Scales are sedentary for most of their lifecycle. They insert flexible stylets into host plants to feed on phloem, xylem, or other tissue. Feeding by scales and other hemipterans reduces plant growth by removing carbohydrates and reducing photosynthesis (Cockfield & Potter, 1990; Cockfield, Potter & Houtz, 1987; Dixon, 1971; Zvereva, Lanta & Kozlov, 2010). Scale insects often reach high populations on urban trees due to factors such as high temperature (Dale & Frank, 2014a, 2014b; Meineke et al., 2013), drought (Dale & Frank, 2017), plant stress (Meineke & Frank, 2018; Speight et al., 1998), and diminished natural enemy communities (Hanks & Denno, 1993; Luck & Dahlstein, 1975; McClure, 1977; Meineke, Dunn & Frank, 2014; Tooker & Hanks, 2000). Our goal was to determine if exotic and native tree species common in urban plantings support similar arthropods, with a specific focus on scale insects and natural enemy communities of arthropod predators and parasitoids (Fig. 1). To achieve this, we examined scale insect and natural enemy abundance on native and exotic tree species in the genera *Quercus* and *Acer*. These genera contain some of the most common trees in urban landscapes (Raupp, Cumming & Raupp, 2006), and native trees in both genera are hosts to many scale species (Frank et al., 2013; Johnson & Lyon, 1976). Our first objective was to compare scale insect abundance on native and exotic species within each genus. Our second objective was to determine if natural enemies of scales and other herbivores were more abundant, diverse, or had different community structure on native or exotic species within each genus.

## Methods

All study trees were located on the grounds of North Carolina State University, an urban campus in Raleigh, NC, USA (35.786°N, 78.672°W). In 2012, we located 10 or 15 trees (Table 1) of each of the following species: *Acer palmatum* (Japanese maple), *A. platanoides* (Norway maple), *A. rubrum* (red maple), *A. saccharum* (sugar maple), *Quercus acutissima* (sawtooth oak), *Q. alba* (white oak), and *Q. phellos* (willow oak). *Acer palmatum* is native to Japan, South Korea, and eastern China. The other exotic maple, *A. platanoides*, is native to eastern Europe and western Russia from Sweden south to Greece. It is also an invasive species that has become established in parts of the eastern and northwestern US and Canada (United States Department of Agriculture (USDA), 2018). *Acer rubrum* is native throughout the eastern half of North America from Quebec, Canada south to Florida, US (United States Department of Agriculture (USDA), 2018). *Acer saccharum* is native throughout much of eastern North America south to North Carolina and Tennessee, US and cool regions further south (United States Department of Agriculture (USDA), 2018). *Quercus acutissima* is native to eastern Asia including China, Korea, and Japan but has become established in some regions of eastern and southern US (United States Department of Agriculture (USDA), 2018). *Quercus alba* is native throughout eastern North America from Quebec, Canada south to Florida and Texas, US (United States Department of Agriculture (USDA), 2018). *Quercus phellos* is native throughout the southern and eastern US north to New Jersey (United States Department of Agriculture (USDA), 2018). North Carolina State University campus is comprised of many land cover types including impervious surfaces, like roads and buildings, lawn and ornamental landscapes, sports fields, and small forest patches. Study trees were in growing in lawn areas surrounded by turf or in mulched landscape beds surrounded by ornamental shrubs.

**Table 1:** 2012 and 2016 scale insect abundance.

2012					
Species	Origin	*n*	Scale insects		BH
*Acer palmatum*	exotic	15	0.92	(0.27)	ab
*Acer platanoides*	exotic	10	0.22	(0.18)	c
*Acer rubrum*	native	10	28.48	(22.33)	a
*Acer saccharum*	native	10	0.22	(0.08)	bc
*Quercus acutissima*	exotic	15	0.33	(0.11)	a
*Quercus alba*	native	15	106.01	(46.00)	b
*Quercus phellos*	native	15	3.55	(1.10)	c

2016					
Species	Origin	*n*	Scale insects		BH
*Acer buergerianum*	exotic	10	0.10	(0.07)	a
*Acer palmatum*	exotic	10	0.15	(0.08)	a
*Acer rubrum*	native	10	65.75	(31.77)	b
*Acer saccharum*	native	10	0.90	(0.31)	c
*Quercus acutissima*	exotic	10	0.30	(0.17)	a
*Quercus alba*	native	10	11.65	(5.24)	b
*Quercus phellos*	native	10	8.75	(3.89)	b

**Note:**

Mean scale insect abundance per 30 cm of branch in 2012 (top) and 2016 (bottom), reported as mean (± standard error) with *n* indicating the number of trees. 2016 counts include live scale insects only. Within each year-genus pair, tree species that share a letter are not different (α = 0.05) based on a Kruskal–Wallis test with a Benjamini–Hochberg (BH) post hoc comparison. Tree species are ordered alphabetically within each year-genus pair.

In October 2012, we pruned five 30 cm branches from each tree and used a dissecting microscope to identify and count scale insects on the branches. Many scale insects, especially those in the family Diaspididae, leave behind waxy covers that can persist on trees after the soft-bodied scale insect has died. In 2012, we counted all scales insects, live or dead, on the branches and identified armored scales (Hemiptera: Diaspididae), soft scales (Hemiptera: Coccidae), and pit scales (Hemiptera: Astrolecaniidae) to family. In May 2012, we used 7.6 × 12.7 cm yellow sticky cards (Great Lakes IPM, Inc., Vestaburg, MI, USA) to capture scale insect natural enemies in the canopy of each tree. Sticky cards were placed in the interior of the canopy, above the first lateral branches, to avoid attracting insects from other areas and incidental capture of insects flying in the vicinity of the trees. Arthropods were sampled for 7–13 days in each tree. We identified common predators to family or order and counted parasitoid wasps without identifying them. One sticky card was lost during sampling.

In 2016, we used ArcMap 10.2.2 (Esri, Redlands, CA, USA) to randomly select 10 study trees of each species from a geo-referenced inventory of trees maintained by the North Carolina State University Facilities Division. We included the same tree species as in 2012 except we replaced the exotic species *A. platanoides*, which was no longer present in sufficient numbers on campus, with another exotic, *A. buergerianum* (trident maple) which is native to eastern Asia. Trees of each species were at least 100 m apart, with the exception of three white oaks (*Q. alba*) which, out of necessity, were at least 50 m apart. From late February through early May 2016, we collected two terminal branches that were at least three m high from each tree to count scale insect abundance. We used a dissecting microscope to identify and count scale insects on the terminal 30 cm of each branch. We used metal probes to determine which scale insects were alive and included only live scale insects in 2016 data analysis. We counted all live scales and identified live armored scales (Hemiptera: Diaspididae), soft scales (Hemiptera: Coccidae), and pit scales (Hemiptera: Astrolecaniidae) to family. We used 7.6 × 12.7 cm yellow sticky cards (Great Lakes IPM, Inc., Vestaburg, MI, USA) to survey natural enemy abundance. We placed a single card in the canopy of each tree for two weeks in June 2016, and we repeated this in July and August 2016. Four sticky cards were lost. We used a dissecting microscope to identify parasitoid wasps to family and to identify common scale insect predators (Goulet & Huber, 1993). Here, we report on the six predator taxa that were collected and identified in both 2012 and 2016. Insect taxa were identified to family (Hemiptera: Anthocoridae, Coleoptera: Carabidae, Coleoptera: Coccinellidae, Diptera: Dolichopodidae) and spiders to order (Araneae).

### Analyses

We conducted all statistical analyses in R version 3.3.2 (R Core Team, 2016) and conducted separate tests for maples and oaks. Because we used different data collection methods in 2012 and 2016, we do not compare results across years. We pooled scale insect counts from all sampled branches on each tree and calculated mean scale insect abundance per 30 cm of branch for use in analyses. To compare scale insect abundance between tree species, we performed four separate Kruskal–Wallis tests, one for each year-genus pair, using R package *agricolae* (De Mendiburu, 2016). We used the Benjamini–Hochberg method for post hoc multiple comparisons when overall tests indicated significant differences (α < 0.05) between tree species (Benjamini & Hochberg, 1995). For each year, we compared total predator and, separately, total parasitoid wasp abundance between tree species using Kruskal–Wallis tests with Benjamini–Hochberg post hoc comparisons as above. For consistency and to account for differences in sampling duration (2012) and lost sticky cards (2016), we converted all predator and parasitoid counts to abundance per 7-day sampling period prior to analysis.

We used R package *mvabund* (Wang et al., 2014) to test whether the abundance of the six predator taxa varied across tree species. *mvabund* fits generalized linear models to multivariate abundance data to test the effects of a predictor variable on both community-level response and the responses of individual taxa (Warton et al., 2015). Distance-based analyses sometimes confound location and dispersion effects, but *mvabund* avoids this problem by allowing the user to specify a mean-variance relationship, for example by using poisson or negative binomial regressions (Warton, Wright & Wang, 2012). We fit negative binomial regressions to each predator taxon using raw counts and offset terms to account for differences in sample durations (oaks in 2012) or numbers (oaks and maples in 2016). To test whether the community of these six predator taxa differed across tree species, we used the *anova.manyglm* function with a Wald test statistic, 1,000 bootstrap iterations, and the default step-down *p*-value adjustment method in *mvabund*. When this test indicated an overall significant effect of tree species, we used the *summary.manyglm* function to test pairwise contrasts of tree species following the methods in Ji et al. (2013) and Bruce (2013). To account for multiple comparisons, we adjusted *p*-values in R’s base package using the Benjamini–Hochberg correction. To obtain univariate statistics for the pairwise comparisons, we used the default step-down *p*-value adjustment method in the *anova.manyglm* function, changing the baseline tree species as necessary.

We used the R package *vegan* (Oksanen et al., 2010) to calculate the Shannon diversity index of the parasitoid wasp communities at the family level. In R’s base package, we used ANOVA to compare the diversity indices between tree species. We used *mvabund* to analyze 2016 parasitoid wasp communities following the same procedures we used for predators. Prior to analysis, we removed parasitoid wasp families that were represented by only a single individual (two families each for maples and oaks).

## Results

### Scale insects

In 2012, we collected 9,757 scale insects, with a mean (± SD) of 21.68 (± 83.74) scale insects per 30 cm of branch per tree. Scale insect abundance varied by tree species for maples (χ^2^ = 15.11, *p* < 0.002), with the native *A. rubrum* having the highest abundance and the exotic *A. platanoides* having the lowest abundance (Table 1). A total of 94.98% of the scales we collected on maples were armored scales (Hemiptera: Diaspididae), and the remaining 5.01% were soft scales (Hemiptera: Coccidae). Differences were also apparent between oak species (χ^2^ = 23.69, *p* < 0.001), with highest abundance on the native *Q. alba* and lowest abundance on the exotic *Q. acutissima* (Table 1). A total of 92.17% of the scales we collected on oaks were armored scales (Diaspididae) 5.39% were pit scales (Hemiptera: Asterolecaniidae), and 1.89% were soft scales (Coccidae).

In 2016, we collected 1,752 live scale insects, with a mean (± SD) of 12.51 (± 43.25) live scale insects per 30 cm of branch per tree. Scale insect abundance was less than in 2012 since we only counted live scales in 2016. Scale abundance varied by tree species for maples (χ^2^ = 17.11, *p* < 0.001) and oaks (χ^2^ = 11.79, *p* < 0.003); both native species of each genus had significantly more scale insects than their exotic congener(s) (Table 1). On maples, 98.87% of the scales we collected were armored scales (Hemiptera: Diaspididae), and the remaining 1.13% were soft scales (Hemiptera: Coccidae). On oaks, 40.58% of the scales we collected were soft scales (Coccidae), 22.46% were pit scales (Hemiptera: Asterolecaniidae), and 21.50% were armored scales (Diaspididae).

### Predators

In 2012, we collected 207 predators in the five predator taxa reported here. Abundance per sample did not differ among maple species (χ^2^ = 2.08, d*f* = 3, *p* = 0.555) or oak species (χ^2^ = 2.27, d*f* = 2, *p* = 0.321) (Table 2). In 2016, we collected 1,074 predators in the five taxa which is more than in 2012 since we sampled three times instead of once. The total number of predators per sample differed among maple species (χ^2^ = 13.22, d*f* = 3, *p* = 0.004), with lower total predator abundance within the exotic *A. palmatum* than in the other maple species (Table 2). There was no difference in total predator abundance among oak species (χ^2^ = 4.02, d*f* = 2, *p* = 0.134) (Table 2).

**Table 2:** 2012 and 2016 predator and parasitoid abundance.

2012								
Species	Origin	*n*	Predators		BH	Parasitoids		BH
*Acer palmatum*	exotic	14	1.93	(1.03)	–	22.36	(4.57)	–
*Acer platanoides*	exotic	10	2.00	(0.77)	–	33.50	(5.93)	–
*Acer rubrum*	native	10	0.90	(0.31)	–	22.90	(3.61)	–
*Acer saccharum*	native	10	0.90	(0.59)	–	30.50	(15.49)	–
*Quercus acutissima*	exotic	15	1.26	(0.29)	–	24.49	(1.77)	a
*Quercus alba*	native	15	1.64	(0.37)	–	42.58	(4.37)	b
*Quercus phellos*	native	15	3.25	(1.16)	–	67.03	(7.27)	c

2016								
Species	Origin	*n*	Predators		BH	Parasitoids		BH
*Acer buergerianum*	exotic	10	2.30	(0.60)	a	30.23	(3.29)	a
*Acer palmatum*	exotic	10	0.72	(0.14)	b	34.87	(4.91)	a
*Acer rubrum*	native	10	3.15	(0.61)	a	34.73	(4.13)	a
*Acer saccharum*	native	10	2.83	(1.32)	a	20.48	(2.13)	b
*Quercus acutissima*	exotic	10	4.86	(1.14)	–	30.25	(5.29)	–
*Quercus alba*	native	10	2.28	(0.31)	–	28.28	(4.36)	–
*Quercus phellos*	native	10	1.98	(0.34)	–	33.63	(3.40)	–

**Note:**

Mean predator and parasitoid wasp abundance per 7-day sample from sticky cards in 2012 (top) and 2016 (bottom), reported as mean (± standard error) with *n* indicating the number of trees. Predator tests were performed separately from parasitoid tests for each year-genus pair. Within each year-genus pair, tree species that share a letter are not different (α = 0.05) based on a Kruskal–Wallis test with a Benjamini–Hochberg (BH) post hoc comparison. Letters are provided only when the overall Kruskal–Wallis test indicated a significant difference between species. Tree species are ordered alphabetically within each year-genus pair.

**Figure 2:**
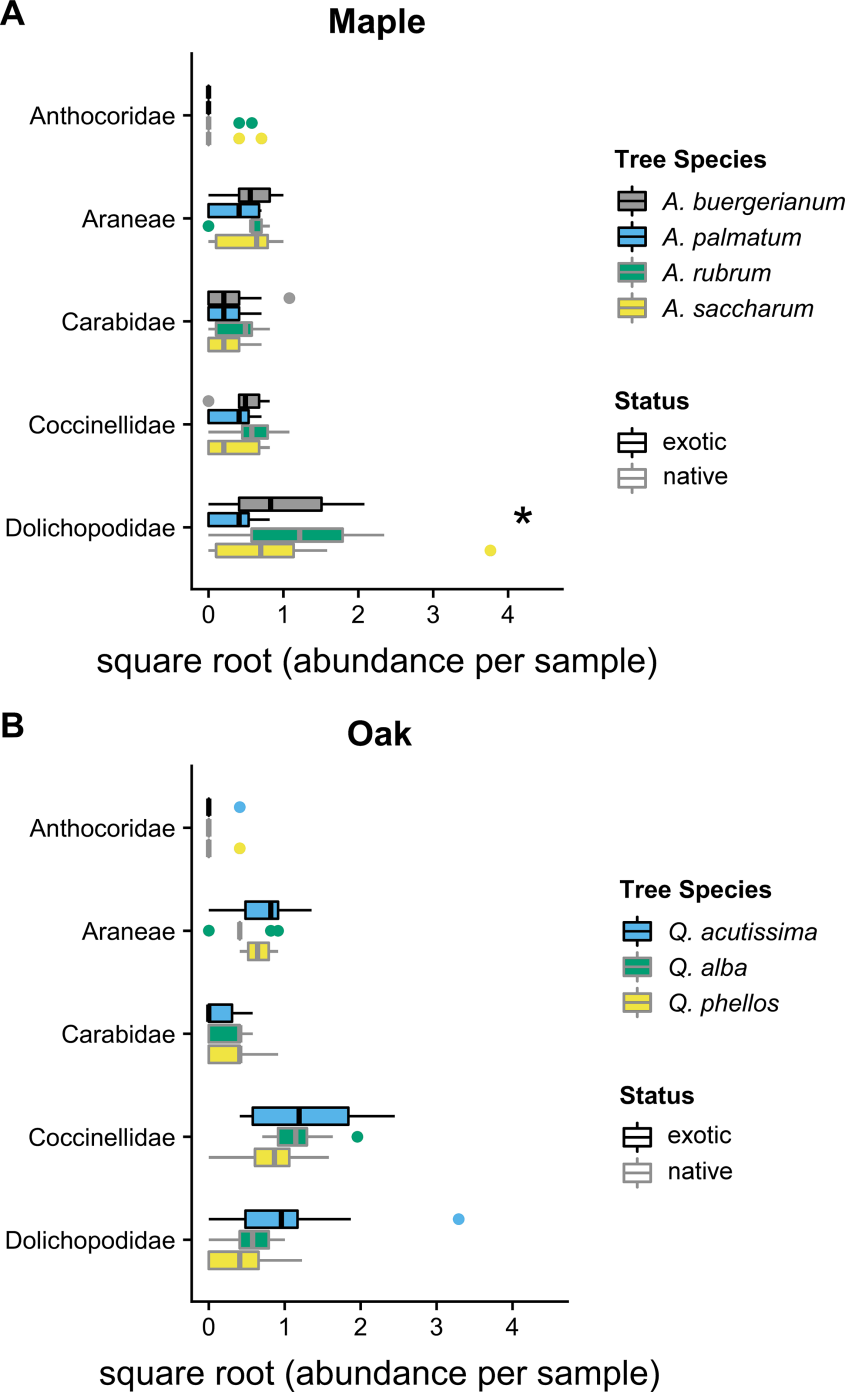
2016 predator communities. Abundance of predators in five taxa in 2016 for (A) maples and (B) oaks. Colored boxes mark the interquartile range (IQR), and whiskers extend to the largest value within 1.5 * IQR. Values beyond 1.5 * IQR are marked as points. Exotic species are outlined in black. Predator taxa that differed significantly between tree species in univariate tests are marked with * (Tables S1 and S2). Global tests indicated significant differences in the predator communities of maples and oaks (*p* < 0.05), where *A. palmatum* was different than other maple species and *Q. acutissima* was different than other oak species (Tables S1 and S2).

In 2012, the composition of predator communities was not different among tree species for maples (Wald statistic = 3.64, *p* = 0.134) or oaks (Wald statistic = 3.92, *p* = 0.074). In 2016, the predator community differed among maple species (Wald statistic = 5.15, *p* < 0.049); the exotic *A. palmatum* had a different community than all other *Acer* species (Fig. 2). This difference was driven by the significantly lower Dolichopodidae abundance within *A. palmatum* (Fig. 2; Table S1). The predator community also differed between oak species in 2016 (Wald statistic = 5.125, *p* = 0.029) (Fig. 2). The exotic *Q. acutissima* had a different overall predator community than the two native species, which was not driven by significant differences in any individual taxa (Fig. 2; Table S2).

### Parasitoid wasps

In 2012, we collected 4,676 parasitoid wasps from 89 samples, with a mean (± SD) of 52.5 (± 47.2) parasitoid wasps per 7-day sample. The number of parasitoid wasps per 7-day sample was not different among maple species (χ^2^ = 4.64, d*f* = 3, *p* = 0.200) but differed among the oak species (χ^2^ = 17.26, d*f* = 2, *p* < 0.001), with highest abundance within a native, *Q. phellos*, and lowest abundance within the exotic, *Q. acutissima* (Table 2). In 2016, we collected 12,520 parasitoid wasps in 25 families from 206 samples. There was a mean (± SD) of 30.35 (± 13.12) parasitoid wasps per 7-day period. The number of parasitoid wasps per sample differed among maple species, with lower total abundance within a native, *A. saccharum*, than the other maple species (χ^2^ = 9.96, d*f* = 3, *p* = 0.019), and did not differ among oak species (χ^2^ = 1.68, d*f* = 2, *p* = 0.431) (Table 2).

**Figure 3:**
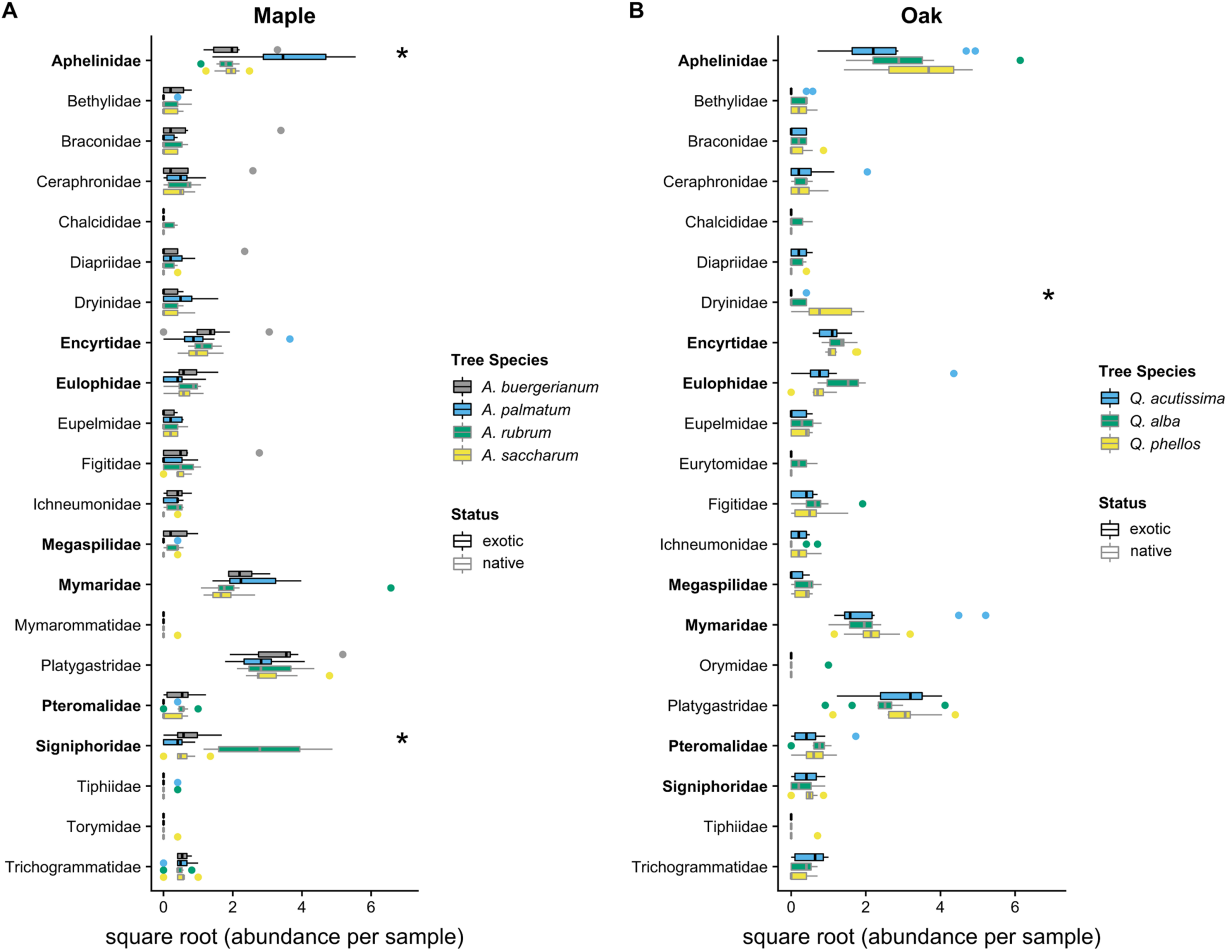
2016 parasitoid wasp communities. Abundance of parasitoid wasps per 7-day sampling period in 2016 on (A) maples and (B) oaks. Colored boxes mark the interquartile range (IQR), and whiskers extend to the largest value within 1.5 * IQR. Values beyond 1.5 * IQR are marked as points. Exotic species are outlined in black. Parasitoid families that differed significantly between tree species in univariate tests are marked with * (Tables S3 and S4). Parasitoid wasp families that use scale insects as hosts (Kosztarab, 1996) are bolded on the *y*-axis. Global tests indicated significant differences in the parasitoid wasp communities of maples and oaks (*p* < 0.05), where all maple species had different communities and *Q. phellos* had a different community than the other two oak species (Tables S3 and S4).

The Shannon diversity index did not differ among maple species (*F*_3,36_ = 2.168, *p* = 0.109) or oak species (*F*_2,27_ = 2.502, *p* = 0.101). Parasitoid community compositions differed among maple species (Wald statistic = 15.33, *p* = 0.001; Fig. 3), with pairwise tests showing differences between each pair of maple species (*p* < 0.05), driven primarily by high Signiphoridae abundance within *A. rubrum* and high Aphelinidae abundance within *A. palmatum* (Table S3). Parasitoid community composition also differed among oak species (Wald statistic = 9.40, *p* = 0.007; Fig. 3), with *Q. phellos* having a different composition than the other two species (Table S4). The abundance of parasitoid wasps in the family Dryinidae was higher on *Q. phellos* than on the other oak species (*p* < 0.05).

## Discussion

Trees are critical for sustaining invertebrate and vertebrate diversity in urban habitats (Smith et al., 2006a, 2006b; Stagoll et al., 2012). Native trees, in particular, are often thought to be critical for supporting local herbivores and the predators and parasitoids that consume them (Goddard, Dougill & Benton, 2010; Isaacs et al., 2009; McKinney, 2002; Tallamy, 2004; Zuefle, Brown & Tallamy, 2007). Thus, increasing the proportion of native tree species is often recommended as a measure to conserve urban animal diversity and reduce homogenization between cities (Alvey, 2006; Clark et al., 1997; Herrmann, Pearse & Baty, 2012; McKinney, 2006). In support of this recommendation and the enemy release hypothesis, native species we sampled in the genera *Acer* and *Quercus* had higher scale insect density than exotic congeners. The scale insect community on maples and oaks in Raleigh includes many species native to the Southeastern US including *Melanaspis tenebricosa*, *M. obscura*, *Parthenolecanium quercifex*, and *Mesolecanium nigrofasciatum*, in addition to exotic species such as *Lopholeucaspis japonica* and *P. corni.* Native *A. rubrum*, *Q. phellos*, and *Q. alba* had scale insect densities two to three orders of magnitude higher than any exotics or the native *A. saccharum*. In terms of supporting native herbivores, and herbivores in general, native tree species in cities are beneficial. However, from the perspective of aesthetics or the ecosystem services (such as carbon fixation) carried out by trees, the densities of scale insects observed on *A. rubrum*, *Q. phellos*, and other urban trees are potentially detrimental. At such densities, scale insects can reduce photosynthesis (Cockfield, Potter & Houtz, 1987), growth (Meineke et al., 2016; Meineke & Frank, 2018; Speight, 1991; Vranjic & Ash, 1997), and the aesthetic or structural condition of trees (Dale & Frank, 2014a; Just, Frank & Dale, 2018; Speight et al., 1998; Sperry et al., 2001). Conversely, in the urban sites we studied, exotic trees had fewer pests and may be more likely to maintain growth and services such as temperature reduction and carbon sequestration with fewer management costs (Chalker-Scott, 2015; Hitchmough, 2011).

Scale insects and other hemipteran herbivores are prey or hosts for hundreds of predatory and parasitic arthropods as well as prey for birds and other vertebrates (Brennan, Morrison & Dahlsten, 2000; Clout & Hay, 1989; Evans, Towns & Beggs, 2015; Moeed & Fitzgerald, 1982; Morse, 1971; Witmer, 1996). In our study, the abundance and community composition of predators and parasitoid wasps, as sampled by sticky cards, were generally similar between native and exotic congeners. In 2012, both predator and parasitoid wasp abundance were highest within the native species *Q. phellos*. Parasitoid wasp abundance was lowest within the exotic species *Q. acutissima*. Parasitoid wasp abundance overall was greatest in *Q. phellos* but the family Dryinidae was the only family that was significantly more abundant. Dryinid wasps parasitize Hemipterans in the suborder Auchenorrhyncha which includes leafhoppers, planthoppers, lace bugs, and others but not scale insects (suborder Sternorrhyncha) (Klejdysz et al., 2018). Oaks support many Auchenorrhyncha that could be hosts for Dryinid wasps including specialists like oak lace bug (*Corythucha arcuata*) and oak treehopper (*Platycotis vittata*) and many generalists (Johnson & Lyon, 1976; Southwood, 1961; Southwood, Moran & Kennedy, 1982; Southwood et al., 2004, 2005). The most abundant parasitoid families within oaks were Aphelinidae, Platygatridae, and Mymaridae. Most Aphelinids are parasitoids of Sternorrhyncha which includes scale insects, aphids, and mealybugs (Viggiani, 1984). *Coccophagus lycimnia* is a common Aphelinid parasitoid of *Parthenolecanium* spp. scale which is the most common scale on *Q. phellos* in our region (Meineke, Dunn & Frank, 2014; Meineke et al., 2013; Robayo Camacho et al., 2018). A total of 21 parasitoid species have been reared from *Parthenolecanium* scales from *Q. phellos* (Meineke, Dunn & Frank, 2014; Meineke et al., 2013; Robayo Camacho et al., 2018). Most Platygastrids parasitize flies in the family Cecidomyiidae many of which are herbivorous leafminers and gall makers but the family also includes predatory flies that prey on scales, aphids, and other Hemipterans (Hagen et al., 1999). All species within the family Mymaridae parasitize eggs of other insects, frequently those of scale insects and their relatives (Hemiptera: Coccoidea) (Harris, 1968).

Where differences in natural enemy abundance were observed within maples in 2016, a native species, *A. saccharum,* had the lowest abundance of predators and parasitoid wasps. The native species *A. rubrum,* which often has severe infestations of gloomy scales (*Melanaspis tenebricosa*) in cities (Dale & Frank, 2014a, 2014b; Metcalf, 1912; Youngsteadt et al., 2015), had significantly higher abundance of Signiphoridae wasps which parasitize scales, mealybugs, or psyllids (Gibson, Huber & Woolley, 1997). *Signiphora* spp. (Signiphoridae), *Encarsia* spp. (Aphelinidae), *Ablerus* spp. (Aphelinidae), and *Coccidoctonus* (Encyrtidae) have been reared from gloomy scales (Dale & Frank, 2014b). *Acer palmatum,* which had very low scale insect density, had significantly more Aphelinidae parasitoids than the other *Acer* species. Members of Aphelinidae, primarily parasitoids of aphids, scales, whiteflies, and other hemipterans, could have been attracted to *A. palmatum* by aphids or other herbivores we did not sample (Frank et al., 2013).

Many other arthropods are present within oak and maple canopies (Johnson & Lyon, 1976; Southwood, Moran & Kennedy, 1982). For example, 537 and 297 lepidopteran species are associated with the genera *Quercus* and *Acer* respectively (Tallamy & Shropshire, 2009). Thus, many parasitoids and predators we captured may interact with herbivores other than scales or with each other as higher-order natural enemies. In addition, sticky cards are a passive sampling technique that is biased toward flying taxa and can capture “tourists” in addition to species that have close associations with tree species or its herbivores. For example, few North American carabid species are arboreal so some of the carabids we captured could have been incidental or tourists in our study trees. However, arthropods collected from *Q. phellos* and *A. rubrum* foliage with sweep nets in previous research included many of the same taxa as collected on sticky cards in addition to predatory mites (Phytosiidae), lacewings (Neuroptera: Coniopterygidae, Chrysopidae), ants (Hymenptera: Formicidae), 17 families of spiders, and many others (McCluney, George & Frank, 2018; Meineke et al., 2017).

Low scale density on *A. palmatum* and other exotic species could have resulted from the combined influence of high natural enemy abundance and low susceptibility to many scale species. Generalist predators in the families Anthocoridae and Coccinellidae, which feed on scales and other Hemipterans, were captured in similar numbers in native and exotic trees. Spiders, which are common in urban trees and feed on many taxa, were also of similar abundance in native and exotic trees (McCluney, George & Frank, 2018; Meineke et al., 2017). Other researchers have also found similar densities of arthropod natural enemies on native and exotic trees even though the exotic species had lower herbivore densities (Hartley, Rogers & Siemann, 2010; Procheş et al., 2008; Southwood, Moran & Kennedy, 1982). Natural enemies frequently visit and remain in habitats due to the vegetation structure, microclimates, or other resources that may be similar for native and exotic tree species. Our results corroborate these findings but this pattern requires further research to understand the factors, other than herbivores, that define the conservation value of exotic trees.

There is growing evidence that some insect taxa, or even insects in general, are declining due to land use change, climate change, exotic plants, insecticides, and other factors (Conrad et al., 2006; Hallmann et al., 2017; Potts et al., 2010). All these issues converge when managing urban trees to conserve arthropods and minimize pests. Urban forest design and planting recommendations generally include increasing tree diversity at multiple taxonomic levels using a mixture of native and non-native species (Raupp, Cumming & Raupp, 2006; Elevitch & Wilkinson, 2001). The primary goal of increasing urban tree diversity has been to reduce catastrophic damage caused by exotic pests such as emerald ash borer and Dutch elm disease. Although we only studied three exotic maples and one exotic oak they are among the most commonly planted species in our region. Our results suggest that planting these exotic tree species is also a valuable contribution to conserving arthropod communities (Chalker-Scott, 2015; Hitchmough, 2011). Since these exotic species are also less susceptible to scales, and potentially other pests, they may not require insecticide applications that harm non-target organisms and thus reverse conservation goals (Goulson, 2013; Luck & Dahlstein, 1975; Raupp et al., 2001; Woodcock et al., 2016).

The exotic species we studied conserved similar arthropod natural enemy abundance and community structure at the family level as their native congeners. There are likely differences in natural enemy communities at lower taxonomic levels particularly among specialists that require a particular herbivore species on which to feed. Native trees, such as oaks that host hundreds of herbivorous species (Southwood, 1961), may have richer specialist herbivore diversity and a different array of natural enemy species than exotic trees (Burghardt et al., 2010; Keane & Crawley, 2002). However, the extent of these differences may vary with arthropod, life stage, feeding guild, taxonomic isolation of the tree species, and other factors we could not assess (Burghardt & Tallamy, 2013, 2015). In addition, arthropod communities on distantly related tree species or those with no native relatives such as ginkos (*Ginkgo biloba*) or crape myrtle (*Lagerstroemi*a spp.) are likely more distinct than comparisons between congeners (Burghardt & Tallamy, 2013, 2015). However, we hypothesize that herbivory and the biodiversity supported by a tree depends as much on urban variables such as habitat fragmentation, impervious surface cover, and temperature as it does on geographic origin of the tree species (Frank, 2014; Le Roux et al., 2018; Long, D’Amico & Frank, 2018; McCluney, George & Frank, 2018; Meineke et al., 2017). Thus, even a native urban tree will likely support a different arthropod community than the same tree species in a natural habitat and have different conservation value (Herrmann, Pearse & Baty, 2012; Manning, Fischer & Lindenmayer, 2006; Turrini & Knop, 2015). Our results support mixing native and exotic trees to achieve conservation and pest management goals.

## Conclusions

Conservation of arthropods, for their own sake, and to support birds are commonly cited reasons to plant native instead of exotic trees in urban spaces (Goddard, Dougill & Benton, 2010; Tallamy, 2004). Our analyses of the scale insect and natural enemy communities in some native and exotic maple and oak species does not fully support this perspective. The native tree species in our research did not always host more herbivorous scale insects than the exotic species and we found similar natural enemy communities within the native and exotic species. Moreover, high densities of scale insects in native *A. rubrum* and *Q. phellos* have been found to reduce tree condition and growth in previous research (Dale & Frank, 2014a; Meineke et al., 2016; Meineke & Frank, 2018). We conclude that the exotic oak and maple species we studied could be as valuable as the native species for conserving arthropod natural enemies. The pest susceptibility of native tree species must be balanced against potential conservation benefits when selecting trees for urban planting.

## Supplemental Information

10.7717/peerj.6531/supp-1 Supplemental Information 1 2016 predator community pairwise comparisons for maples. Pairwise comparisons of predator communities (for five taxa) on maples in 2016. p values for overall pairwise tests were adjusted using the Benjamini-Hochberg method (BH). Univariate p values were adjusted using the standard step-down resampling procedure in *mvabund*. Acronyms identifying exotic tree species are bolded. (ACBU: *A. buergerianum*, ACPA: *A. palmatum*, ACRU: *A. rubrum*, ACSA: *A. saccharum*).

10.7717/peerj.6531/supp-2 Supplemental Information 2 2016 predator community pairwise comparisons for oaks. Pairwise comparisons of predator communities (for five taxa) on oaks in 2016. p values for overall pairwise tests were adjusted using the Benjamini-Hochberg method (BH). Univariate p values were adjusted using the standard step-down resampling procedure in *mvabund*. Acronyms identifying exotic tree species are bolded. (QUAC: *Q. acutissima*, QUAL: *Q. alba*, QUPH: *Q. phellos*).

10.7717/peerj.6531/supp-3 Supplemental Information 3 2016 parasitoid wasp community pairwise comparisons for maples. Pairwise comparisons of parasitoid communities on maples in 2016. p values for overall tests were adjusted using the Benjamini-Hochberg method (BH). Univariate p values were adjusted using the standard step-down resampling procedure in *mvabund*. A dash indicates that parasitoid abundance was not high enough for a particular pairwise comparison. Acronyms identifying exotic tree species are bolded. (ACBU: *A. buergerianum*, ACPA: *A. palmatum*, ACRU: *A. rubrum*, ACSA: *A. saccharum*).

10.7717/peerj.6531/supp-4 Supplemental Information 4 2016 parasitoid wasp community pairwise comparisons for oaks. Pairwise comparisons of parasitoid communities on oaks in 2016. p values for overall tests were adjusted using the Benjamini-Hochberg method (BH). Univariate p values were adjusted using the standard step-down resampling procedure in *mvabund*. A dash indicates that parasitoid abundance was not high enough for a particular pairwise comparison. In the global univariate test, Eulophidae did not show a significant difference across tree species (p > 0.05; data not shown) and thus is not marked with an asterisk on Figure 2. Acronyms identifying exotic tree species are bolded. (QUAC: *Q. acutissima*, QUAL: *Q. alba*, QUPH: *Q. phellos*).

## Additional Information and Declarations

### Data Availability

The following information was supplied regarding data availability: Frank, Steven; Backe, Kristi (2018): Exotic urban trees conserve similar natural enemy communities to native congeners but have fewer pests. figshare. Fileset. DOI 10.6084/m9.figshare.7243181.v1.
