# Supplementary material for: Exotic urban trees conserve similar natural enemy communities to native congeners but have fewer pests
Source: PeerJ. 2019 Mar 7;7:e6531. doi: 10.7717/peerj.6531 (PMC6409088; doi:10.7717/peerj.6531)
Supplement: Supplemental Information 1 — Pairwise comparisons of predator communities (for five taxa) on maples in 2016. p values for overall pairwise tests were adjusted using the Benjamini-Hochberg method (BH). Univariate p values were adjusted using the standard step-down resampling procedure in mvabund. Acronyms identifying exotic tree species are bolded. (ACBU: A. buergerianum, ACPA: A. palmatum, ACRU: A. rubrum, ACSA: A. saccharum). [file peerj-07-6531-s001.docx]

|  | **ACBU** and **ACPA** | | **ACBU** and ACRU | | **ACBU** and ACSA | |
| --- | --- | --- | --- | --- | --- | --- |
|  | Wald statistic | p value | Wald statistic | p value | Wald statistic | p value |
| Overall | 3.61 | **0.032** (BH) | 1.42 | 0.815 (BH) | 1.35 | 0.815 (BH) |
|  | | | | | | |
| Anthocoridae | - | - | 0.04 | 0.871 | 0.04 | 0.93 |
| Araneae | 1.62 | 0.331 | 0.57 | 0.871 | 0.34 | 0.93 |
| Carabidae | 0.76 | 0.529 | 0.34 | 0.871 | 0.76 | 0.93 |
| Coccinellidae | 1.42 | 0.331 | 1.17 | 0.669 | 0.75 | 0.93 |
| Dolichopodidae | 3.19 | **0.025** | 0.69 | 0.871 | 0.57 | 0.93 |
|  | | | | | | |
|  | **ACPA** and ACRU | | **ACPA** and ACSA | | ACRU and ACSA | |
|  | Wald statistic | p value | Wald statistic | p value | Wald statistic | p value |
| Overall | 4.43 | **0.024** (BH) | 3.68 | **0.024** (BH) | 2.19 | 0.707 (BH) |
|  | | | | | | |
| Anthocoridae | 0.04 | 0.452 | 0.04 | 0.941 | 0.26 | 0.987 |
| Araneae | 1.50 | 0.241 | 1.25 | 0.500 | 0.13 | 0.988 |
| Carabidae | 1.42 | 0.241 | 0.00 | 0.941 | 1.42 | 0.472 |
| Coccinellidae | 2.22 | 0.089 | 0.49 | 0.883 | 1.54 | 0.472 |
| Dolichopodidae | 3.99 | **0.006** | 3.08 | 0.054 | 0.01 | 0.990 |
