# Supplementary material for: Exotic urban trees conserve similar natural enemy communities to native congeners but have fewer pests
Source: PeerJ. 2019 Mar 7;7:e6531. doi: 10.7717/peerj.6531 (PMC6409088; doi:10.7717/peerj.6531)
Supplement: Supplemental Information 2 — Pairwise comparisons of predator communities (for five taxa) on oaks in 2016. p values for overall pairwise tests were adjusted using the Benjamini-Hochberg method (BH). Univariate p values were adjusted using the standard step-down resampling procedure in mvabund. Acronyms identifying exotic tree species are bolded. (QUAC: Q. acutissima, QUAL: Q. alba, QUPH: Q. phellos). [file peerj-07-6531-s002.docx]

|  | **QUAC** and QUAL | | **QUAC** and QUPH | | QUAL and QUPH | |
| --- | --- | --- | --- | --- | --- | --- |
|  | Wald statistic | p value | Wald statistic | p value | Wald statistic | p value |
| Overall | 4.12 | **0.018** (BH) | 4.19 | **0.018** (BH) | 2.11 | 0.407 (BH) |
|  | | | | | | |
| Anthocoridae | 0.04 | 0.520 | 0.54 | 0.339 | 0.04 | 0.935 |
| Araneae | 2.24 | 0.128 | 1.09 | 0.339 | 1.84 | 0.292 |
| Carabidae | 1.08 | 0.520 | 1.54 | 0.295 | 0.74 | 0.724 |
| Coccinellidae | 0.90 | 0.520 | 1.89 | 0.231 | 1.52 | 0.401 |
| Dolichopodidae | 2.98 | 0.086 | 2.66 | 0.116 | 0.09 | 0.935 |
