# Supplementary material for: Exotic urban trees conserve similar natural enemy communities to native congeners but have fewer pests
Source: PeerJ. 2019 Mar 7;7:e6531. doi: 10.7717/peerj.6531 (PMC6409088; doi:10.7717/peerj.6531)
Supplement: Supplemental Information 3 — Pairwise comparisons of parasitoid communities on maples in 2016. p values for overall tests were adjusted using the Benjamini-Hochberg method (BH). Univariate p values were adjusted using the standard step-down resampling procedure in mvabund. A dash indicates that parasitoid abundance was not high enough for a particular pairwise comparison. Acronyms identifying exotic tree species are bolded. (ACBU: A. buergerianum, ACPA: A. palmatum, ACRU: A. rubrum, ACSA: A. saccharum). [file peerj-07-6531-s003.docx]

|  | **ACBU** and **ACPA** | | **ACBU** and ACRU | | **ACBU** and ACSA | |
| --- | --- | --- | --- | --- | --- | --- |
|  | Wald statistic | p value | Wald statistic | p value | Wald statistic | p value |
| Overall | 9.56 | **0.002** (BH) | 7.42 | **0.005** (BH) | 7.16 | **0.004** (BH) |
|  | | | | | | |
| Aphelinidae | 4.24 | **0.009** | 0.90 | 0.992 | 0.28 | 0.978 |
| Bethylidae | 2.25 | 0.498 | 0.84 | 0.992 | 1.44 | 0.808 |
| Braconidae | 2.84 | 0.281 | 2.07 | 0.726 | 2.80 | 0.276 |
| Ceraphronidae | 1.13 | 0.863 | 0.73 | 0.992 | 1.58 | 0.808 |
| Chalcididae | - | - | 0.04 | 0.999 | - | - |
| Diapriidae | 1.22 | 0.863 | 2.15 | 0.726 | 2.34 | 0.437 |
| Dryinidae | 2.61 | 0.327 | 0.05 | 0.999 | 0.60 | 0.963 |
| Encyrtidae | 0.27 | 0.964 | 1.39 | 0.957 | 1.61 | 0.808 |
| Eulophidae | 1.36 | 0.863 | 0.03 | 0.999 | 0.77 | 0.963 |
| Eupelmidae | 1.40 | 0.863 | 0.89 | 0.992 | 0.65 | 0.963 |
| Figitidae | 1.86 | 0.694 | 1.11 | 0.979 | 1.94 | 0.686 |
| Ichneumonidae | 0.96 | 0.863 | 0.50 | 0.992 | 2.38 | 0.413 |
| Megaspilidae | 2.25 | 0.498 | 1.04 | 0.989 | 2.25 | 0.480 |
| Mymaridae | 1.26 | 0.863 | 0.65 | 0.992 | 3.01 | 0.174 |
| Mymarommatidae | - | - | - | - | 0.04 | 0.978 |
| Platygastridae | 1.72 | 0.737 | 0.64 | 0.992 | 0.80 | 0.963 |
| Pteromalidae | 2.83 | 0.281 | 0.28 | 0.996 | 1.75 | 0.737 |
| Signiphoridae | 2.56 | 0.337 | 5.71 | **0.002** | 1.18 | 0.889 |
| Tiphiidae | 0.04 | 0.964 | 0.04 | 0.999 | - | - |
| Torymidae | - | - | - | - | 0.04 | 0.978 |
| Trichogrammatidae | 0.26 | 0.964 | 1.30 | 0.961 | 0.18 | 0.978 |
|  | | | | | | |
|  | **ACPA** and ACRU | | **ACPA** and ACSA | | ACRU and ACSA | |
|  | Wald statistic | p value | Wald statistic | p value | Wald statistic | p value |
| Overall | 11.29 | **0.002** (BH) | 7.80 | **0.002** (BH) | 8.32 | **0.002** (BH) |
|  | | | | | | |
| Aphelinidae | 5.47 | **0.002** | 5.01 | **0.002** | 0.90 | 0.963 |
| Bethylidae | 1.64 | 0.786 | 1.47 | 0.859 | 0.48 | 0.993 |
| Braconidae | 1.40 | 0.885 | 0.38 | 0.977 | 1.17 | 0.920 |
| Ceraphronidae | 0.66 | 0.984 | 0.78 | 0.972 | 1.53 | 0.748 |
| Chalcididae | 0.04 | 0.984 | - | - | 0.04 | 0.993 |
| Diapriidae | 1.70 | 0.780 | 2.09 | 0.529 | 0.95 | 0.960 |
| Dryinidae | 2.64 | 0.254 | 2.01 | 0.578 | 0.63 | 0.986 |
| Encyrtidae | 0.70 | 0.984 | 0.94 | 0.972 | 0.53 | 0.993 |
| Eulophidae | 1.67 | 0.786 | 0.88 | 0.972 | 1.05 | 0.957 |
| Eupelmidae | 0.49 | 0.984 | 0.82 | 0.972 | 0.30 | 0.993 |
| Figitidae | 1.12 | 0.948 | 0.58 | 0.972 | 0.96 | 0.960 |
| Ichneumonidae | 0.47 | 0.984 | 1.75 | 0.694 | 2.08 | 0.599 |
| Megaspilidae | 1.92 | 0.632 | 0.00 | 0.977 | 1.92 | 0.615 |
| Mymaridae | 0.14 | 0.984 | 3.34 | 0.059 | 2.06 | 0.615 |
| Mymarommatidae | - | - | 0.04 | 0.977 | 0.04 | 0.993 |
| Platygastridae | 1.13 | 0.948 | 1.02 | 0.969 | 0.14 | 0.993 |
| Pteromalidae | 2.89 | 0.164 | 1.81 | 0.665 | 1.93 | 0.615 |
| Signiphoridae | 7.83 | **0.001** | 1.46 | 0.865 | 6.83 | **0.002** |
| Tiphiidae | 0.00 | 0.984 | 0.04 | 0.977 | 0.04 | 0.993 |
| Torymidae | - | - | 0.04 | 0.977 | 0.04 | 0.993 |
| Trichogrammatidae | 0.95 | 0.963 | 0.36 | 0.977 | 1.27 | 0.887 |
