# Supplementary material for: Exotic urban trees conserve similar natural enemy communities to native congeners but have fewer pests
Source: PeerJ. 2019 Mar 7;7:e6531. doi: 10.7717/peerj.6531 (PMC6409088; doi:10.7717/peerj.6531)
Supplement: Supplemental Information 4 — Pairwise comparisons of parasitoid communities on oaks in 2016. p values for overall tests were adjusted using the Benjamini-Hochberg method (BH). Univariate p values were adjusted using the standard step-down resampling procedure in mvabund. A dash indicates that parasitoid abundance was not high enough for a particular pairwise comparison. In the global univariate test, Eulophidae did not show a significant difference across tree species (p > 0.05; data not shown) and thus is not marked with an asterisk on Figure 2. Acronyms identifying exotic tree species are bolded. (QUAC: Q. acutissima, QUAL: Q. alba, QUPH: Q. phellos). [file peerj-07-6531-s004.docx]

|  | **QUAC** and QUAL | | **QUAC** and QUPH | | QUAL and QUPH | |
| --- | --- | --- | --- | --- | --- | --- |
|  | Wald statistic | p value | Wald statistic | p value | Wald statistic | p value |
| Overall | 5.62 | 0.064 (BH) | 7.39 | **0.003** (BH) | 6.06 | **0.027** (BH) |
|  | | | | | | |
| Aphelinidae | 0.86 | 0.968 | 1.36 | 0.895 | 0.53 | 0.995 |
| Bethylidae | 0.93 | 0.966 | 1.38 | 0.895 | 0.66 | 0.995 |
| Braconidae | 0.28 | 0.998 | 0.65 | 0.969 | 0.42 | 0.995 |
| Ceraphronidae | 1.93 | 0.744 | 1.27 | 0.915 | 0.63 | 0.995 |
| Chalcididae | 0.04 | 0.998 | - | - | 0.04 | 0.998 |
| Diapriidae | 1.28 | 0.874 | 1.79 | 0.679 | 0.89 | 0.989 |
| Dryinidae | 0.76 | 0.975 | 4.29 | **0.012** | 4.43 | **0.004** |
| Encyrtidae | 1.43 | 0.826 | 0.74 | 0.969 | 0.64 | 0.995 |
| Eulophidae | 0.20 | 0.998 | 2.46 | 0.500 | 4.32 | **0.007** |
| Eupelmidae | 1.49 | 0.820 | 0.89 | 0.969 | 0.82 | 0.989 |
| Eurytomidae | 0.05 | 0.998 | - | - | 0.05 | 0.998 |
| Figitidae | 2.19 | 0.611 | 1.84 | 0.679 | 0.20 | 0.998 |
| Ichneumonidae | 0.36 | 0.998 | 1.03 | 0.958 | 1.11 | 0.969 |
| Megaspilidae | 2.37 | 0.491 | 1.50 | 0.854 | 1.11 | 0.969 |
| Mymaridae | 1.81 | 0.744 | 0.89 | 0.969 | 1.48 | 0.873 |
| Orymidae | 0.05 | 0.998 | - | - | 0.04 | 0.998 |
| Platygastridae | 1.15 | 0.923 | 0.19 | 0.984 | 1.33 | 0.915 |
| Pteromalidae | 0.27 | 0.998 | 0.08 | 0.984 | 0.26 | 0.998 |
| Signiphoridae | 0.52 | 0.993 | 0.35 | 0.984 | 0.36 | 0.998 |
| Tiphiidae | - | - | 0.04 | 0.984 | 0.04 | 0.998 |
| Trichogrammatidae | 1.81 | 0.744 | 2.69 | 0.354 | 1.25 | 0.936 |
